# Corona! Die Krise der Verschlankung und ihre Folgen

**DOI:** 10.1007/s41449-020-00216-9

**Published:** 2020-06-19

**Authors:** Irene Raehlmann

**Affiliations:** 1 Hainstr. 20 A, 96047 Bamberg, Deutschland; 2 Otto-Friedrich-Universität Bamberg, Bamberg, Deutschland

## Einleitung

Die Corona-Pandemie ist eine Gefahr für die Gesundheit und das Leben der Menschheit, deren problematische Folgen sich in allen gesellschaftlichen Bereichen und im Alltag niederschlagen. Die rasante, weltweite Ausbreitung des Corona-Virus ist allem Anschein nach auch der Globalisierung der letzten Jahrzehnte geschuldet: Unternehmen haben ihre Arbeit global verteilt und arbeiten international eng verflochten zusammen, der wachsende Tourismus hat mittlerweile den gesamten Globus im Blick. In Reaktion auf die Katastrophe kommt es zu umfassenden staatlichen Interventionen, die die zahlreichen Facetten der Krise zu mildern versuchen und dabei sogar Grundrechte der BürgerInnen außer Kraft setzen. Im Folgenden geht es um die gravierenden Auswirkungen auf die Wirtschaft mit ihren diversen Sektoren und zahlreichen Branchen. Diese soll durch umfangreiche staatliche Finanzhilfen gestützt werden, aber dennoch gilt eine Rezession als sicher und eine Weltwirtschaftskrise als wahrscheinlich. Allerdings sind die entwickelten Wirtschaftsgesellschaften nicht nur mit einer Naturkatastrophe und ihren Auswirkungen konfrontiert, sondern diese ist in weitere, schon länger schwelende Krisen eingebettet wie dem Austritt GB aus der EU, dem Handelskrieg zwischen den USA und China, dem immer wieder aufflammenden handelspolitischen Streit der USA mit Europa sowie last not least mit dem Klimawandel. Ferner durchlaufen die Industriebranchen mit der Digitalisierung und der Forderung nach umweltverträglichen Produkten und einer entsprechenden Fertigung einen fundamentalen Transformationsprozess. Für Deutschland kommt außer diesen Verwerfungen noch eine weitere Herausforderung hinzu, nämlich als „Exportweltmeister“ international auf Märkten überaus machtvoll präsent zu sein – eine durchaus ambivalente Meisterschaft, wie der Bundesverband der deutschen Industrie (BDI) angesichts der Pandemie jüngst feststellte: „Es werde deutlich, ‚wie verletzlich die exportorientierte und international arbeitsteilig organisierte Wirtschaft ist‘“ (zitiert nach: Busse 2020, S. 1). Hinzu kommt, dass eine Weltwirtschaftskrise die deutschen Unternehmen besonders treffen würde, da deren Güter und Dienstleistungen nicht mehr im gewohnten Umfang aus dem Ausland nachgefragt würden. Diese nachteilige Aussicht würde sich weiter zuspitzen, falls die europäischen Nachbarländer, die Hauptabnehmer deutscher Exporte, die Krise nicht überwinden (können).

In den letzten drei Jahrzehnten hat sich ein Organisations- und Arbeitsmodell durchgesetzt, das von den privaten und öffentlichen Akteuren in der Produktion, in den Dienstleistungen und in der Verwaltung zum Leitbild erkoren wurde. Es avancierte zu einem generellen Muster für die Gestaltung von Organisationen. Die Innovation diffundierte jedoch weltweit erst durch eine weitere, nämlich technische. Beide Neuerungen zusammen beschleunigen den Wandel, der vor allem durch stete technologische Fortschritte immer schneller wird. Das Modell firmiert unter dem Namen lean production bzw. lean administration. Es intendiert, eine möglichst schlanke, von allem vermeintlich überflüssigen Ballast bereinigte Organisation zu etablieren, wobei die Informationstechnologie, vornehmlich in Gestalt des Internets als Fundament für die globale Reichweite und die ständige Temposteigerung gilt. Diese zum Ideal stilisierte Organisation ist durch die Corona-Pandemie in eine Krise gestürzt. Sie wird vermutlich überleben, sofern tiefgreifende Korrekturen erfolgen. Zu erinnern ist daran, dass dieses System angesichts augenfälliger Dysfunktionen in den letzten Jahren bereits verändert wurde.

Das Thema entfalte ich in drei Schritten: Zunächst wird das Lean-Konzept vorgestellt, sodann werden diverse Dysfunktionen und mögliche Korrekturen diskutiert und abschließend für ein erweitertes Verständnis von Rationalisierung geworben.

## Lean production/lean administration – ein Rationalisierungsmodell für das 21. Jahrhundert?

Im Zuge der Durchsetzung moderner Gesellschaften wurde Rationalisierung zu einem zentralen Thema. Diesbezügliche Prozesse, die sich in der Lebensführung der Menschen und in den gesellschaftlichen Organisationen manifestierten, wurden überwiegend positiv aufgenommen. Mit der Industrialisierung und der zunehmenden Gründung von Unternehmen bestimmten technisch-organisatorische Rationalisierungen die Praxis des Managements. Mit Blick auf deren Folgen zeigt sich die Ambivalenz dieser Maßnahmen: Während die Arbeitgeber die gestiegene Effizienz und Wirtschaftlichkeit sowie geringere Kosten begrüßen und sich mithin ihre Gewinnerwartungen erhöhen, anerkennen die Arbeitskräfte zwar die effizientere Arbeitsgestaltung mit reduzierter Beanspruchung, gleichzeitig aber nimmt diese durch eine Intensivierung der Arbeit zu, die zu Verschleiß und sogar Erwerbslosigkeit führen kann, sofern die Rationalisierung nicht mit einem Wirtschaftswachstum bzw. einem Nachfrageschub einhergeht. Die idealtypische, widersprüchliche Konstellation – Arbeitskräfte, Arbeitgeber bzw. Management und Gesellschaft – kennzeichnet das unternehmerische Rationalisierungshandeln, wiewohl auch eine teilweise Übereinstimmung der Interessen nicht auszuschließen ist.

Diese Ambivalenz kennzeichnet auch die jüngeren Ansätze, wobei bei aller Kritik festzuhalten ist, dass sowohl die Wissenschaftliche Betriebsführung bzw. der Taylorismus als auch das aktuelle, ebenfalls global einflussreiche Modell lean production Grundlage der Massenproduktion sind, die es ermöglicht, weltweit Güter anzubieten, die von den Menschen bzw. den Konsumenten für attraktiv und begehrenswert erachtet werden. Konsumgesellschaften, wie moderne Gesellschaften häufig apostrophiert werden, wären ohne die Massenproduktion kaum vorstellbar. Allerdings hat in jüngerer Zeit, vor allem in den westlichen Industriegesellschaften ein Individualisierungsprozess stattgefunden, der sich auch in einem veränderten Kaufverhalten widerspiegelt, d. h. die Konsumenten wollen ihre individuellen Bedürfnisse und Wünsche stärker berücksichtigt sehen. Darauf muss die Produktion eingehen und ein differenziertes Angebot, etwa beim Erscheinungsbild der Waren, offerieren, ohne dass die Vorteile der Massenproduktion, nämlich gemeinsame grundlegende Komponenten, beispielsweise technischer Natur, aus dem Blick geraten.

Beide Ansätze zählen zu den Rationalisierungskonzepten, die die Organisations- und Arbeitsgestaltung maßgeblich prägten und prägen. Typisch für sie ist, dass sie mit dem Anspruch eines one best way auftreten, sie erscheinen mithin als alternativlos mit Blick auf das wirtschaftliche Optimum. Beide Systeme lösen sich nicht einfach ab, sondern existieren gleichzeitig, d. h. der Taylorismus überlebt, obwohl er von wissenschaftlich wie praktisch Tätigen längst für obsolet erklärt wurde. Schließlich waren und sind für beide Ansätze Produktionsunternehmen wie die Automobilbranche erste Experimentier- und Einsatzfelder, allerdings weitete und weitet sich die Umsetzung weltweit auf alle Sektoren und Branchen aus, die private Lebenswelt bleibt ebenfalls davon nicht unberührt. Es kann nicht erstaunen, dass sich die Rationalisierungskonzepte im Verlauf eines Jahrhunderts grundlegend gewandelt haben. Handelte es sich früher um punktuelle, durchaus verbundene Rationalisierungen, so beabsichtigen die diesbezüglichen Anstrengungen heutzutage, vor allem befeuert durch die Informationstechnologie wie dem Internet, einen ganzheitlichen, systemischen Zugriff, der betriebliche und außerbetriebliche Dimensionen gleichermaßen zu erfassen versucht. Schließlich haben international vergleichende Untersuchungen, die in den letzten Jahrzehnten forciert wurden, offengelegt, dass die Umsetzung von Arbeits- und Organisationsmodellen nicht gemäß dem idealtypischen Konstrukt erfolgt, sondern von spezifischen nationalen, sozio-kulturellen Besonderheiten wie etwa dem Berufsbildungssystem und dem System der industriellen Beziehungen geprägt wird (vgl. Lutz 1986, S. 34 ff.). Demzufolge gilt Gestaltung als ein pfadabhängiger Prozess.

Lean Production, das in den Grundzügen von dem Japaner Taiichi Ohno (1978/1993) entwickelt und als Toyota-Produktionssystem bekannt wurde, errang Anfang der neunziger Jahre des vergangenen Jahrhunderts weltweit Popularität. Der Erfolg gründete auf der international vergleichenden Studie des Massachusetts Institute of Technology (MIT) über „Die zweite Revolution in der Autoindustrie“ (vgl. Womack et al. 1990/1994) und avancierte zu einem globalen Bestseller. Der programmatische Titel erinnert an die erste, von Henry Ford und Frederick Taylor initiierte Umwälzung im Automobilbau. Symbol der damaligen Revolution war das Fließband verbunden mit einer extremen Arbeitsteilung. Lean Production wurde zum Leitbild von Unternehmensberatern und Rationalisierungsexperten und versprach eine ultimative Lösung wirtschaftlicher Probleme. „Die Euphorie (…) löste in den westlichen Industriegesellschaften eine der größten Rationalisierungswellen der Nachkriegszeit aus“ (Neuhaus 2013, S. 17). Die Protagonisten aus dem MIT stellten in Aussicht: „Am Ende (…) wird die schlanke Produktion die Massenproduktion und die verbliebenen Vertreter der handwerklichen Fertigung in allen Bereichen industrieller Betätigung ersetzen, um das weltweite Standardproduktionssystem des einundzwanzigsten Jahrhunderts zu werden. Diese Welt wird völlig anders und sehr viel besser sein“ (Womack et al. 1994, S. 291 f.).

Die globale Ausstrahlung ist zweifellos auch der Tatsache geschuldet, dass sich das System in das weltweit vorherrschende Wirtschaftsmodell problemlos einfügte. Es war anschlussfähig, wobei realistischerweise von Wechselwirkungen auszugehen ist. Mit dem Niedergang der realsozialistischen Wirtschaftsgesellschaften avancierte nämlich das neoklassische bzw. neoliberale Modell, das in den USA und GB bereits seit Ende der siebziger Jahre die Agenda bestimmte, zum universellen Vorbild wirtschaftlichen Handelns. Es hat bis heute mehr oder weniger Bestand, obwohl spätestens seit der Finanzkrise 2008/09 seine Legitimation erschüttert ist. Die schlanke Fabrik sollte ermöglichen, die Kosten zu minimieren und den Gewinn zu maximieren. Die Politik der Verschlankung wurde gleichermaßen typisch für das staatliche Handeln, sodass ergänzend von lean administration gesprochen wird. Mittlerweile versuchen Akteure, diesen Prinzipien bei der organisatorischen Gestaltung allgemeine Geltung zu verschaffen. Nach betriebswirtschaftlichen Kriterien wurden beispielsweise öffentliche Dienstleistungen, die die Grundbedürfnisse der BürgerInnen stillen sollen, bewertet, und so kam es auch hier zur Verschlankung. In den Bereichen Wohnen, Infrastruktur, Sicherheit, Gesundheit und Bildung kam es ferner zu Privatisierungen und im Zuge dessen wurde ebenfalls Personal eingespart und Ressourcen abgebaut, was für die verbliebenen Beschäftigten unübersehbar eine Arbeitsintensivierung impliziert(e).

Was bedeutet lean production in der betrieblichen Realität? Dieses Produktionssystem, so die MIT-Wissenschaftler, sei schlank, weil es „von allem weniger einsetzt als die Massenfertigung – die Hälfte des Personals in der Fabrik, die Hälfte der Produktionsfläche, die Hälfte der Investition in Werkzeuge, die Hälfte der Zeit für die Entwicklung eines neuen Produktes. Sie erfordert auch weit weniger als die Hälfte des notwendigen Lagerbestands, führt zu viel weniger Fehlern“ (ebd., S. 19). Die Ziele solcher Unternehmen seien „kontinuierlich sinkende Preise, Null Fehler, keine Lagerbestände und beliebige Produktvielfalt“ (ebd., S. 20).

Im Kontext der Entwicklung schlanker Unternehmen wurden und werden verschiedene Methoden eingesetzt: (1.) Outsourcing, also Aus- bzw. Verlagerung einzelner Tätigkeits- und ganzer Geschäftsbereiche, etwa Fertigung, Buchhaltung sowie Forschung und Entwicklung. Das Internet ermöglicht und beschleunigt die Internationalisierung, wiewohl auch national Ausgründungen erfolgen. Verantwortlich dafür sind in erster Linie die unterschiedlichen Entgelte zwischen einzelnen Ländern. (2.) Intendiert wird eine weltweite, kostengünstigste Beschaffung und (3.) eine Beschaffung von möglichst nur einem Lieferanten. (4.) Die Produktion wird durchgängig nach dem Prinzip just-in-time organisiert, d. h. die Nachfrage bestimmt die Produktion, der Stand der Produktion bestimmt den Zeitpunkt der Lieferung von Vorprodukten, so dass sich eine kostspielige Lagerhaltung erübrigt.

Schon im Verlauf der Umsetzung dieses Systems seit den neunziger Jahren zeigten sich Probleme, die den Erfolg in Frage stellten. Dabei ist die strikte Kostenorientierung nicht der kritische Punkt, aber deren Priorisierung ohne ausreichende Rücksicht auf die Folgen. In der Corono-Pandemie erreichen diese Fehlrationalisierungen ein Ausmaß, das den Kampf gegen die Krise schwächt, bisweilen sogar verhindert und mithin deren Ausweitung sogar begünstigen kann.

## Dysfunktionale Folgen der Lean-Modelle und Wege aus dem Dilemma

In der derzeitigen Katastrophe zeigen sich die dysfunktionalen Folgen von lean production und lean administration in aller Schärfe. Dennoch verbietet es sich, Probleme bei der Bewältigung der Krise sozusagen monokausal, allein mit solchen Dysfunktionen erklären zu wollen. Im Unterschied zu früheren Wirtschaftskrisen haben wir es mit einem Angebots- und einem Nachfrageschock gleichzeitig zu tun, die Rezessionen national wie international auslösen, wobei deren Tiefe und Dauer nicht zu prognostizieren sind. Kenneth Rogoff (2020, S. 24), früherer Chefökonom des Internationalen Währungsfonds (IWF), erklärte diesen nicht vorhersehbaren „Doppelschock“ in einem Interview so: „Politiker haben geglaubt, dass die größte Gefahr von einem Nachfrageschock ausgehen würde, wenn also die Menschen weniger kaufen und konsumieren. Aber wenn die Menschen wegen Corona nicht arbeiten gehen können, dürfen und wollen, dann können Lieferketten zusammenbrechen, Waren werden knapp, Preise steigen. Das ist dann der Angebotsschock, den man nicht abfedern kann, indem man die Nachfrage stützt.“ Die Komplexität der Krise lässt sich am Beispiel der Wirtschaftsbeziehungen zwischen Deutschland und China veranschaulichen. China ist nach den EU-Ländern der wohl wichtigste Handelspartner für die deutsche Industrie. Die Wochenzeitung „Die Zeit“, die eine Umfrage unter den Dax-Unternehmen durchführte, konnte zeigen: „Fast alle der 30 Konzerne sind in irgendeiner Art vom Ausbruch des Coronavirus betroffen (…). Sei es, weil Produktionsstätten vorübergehend geschlossen sind, sei es, weil einzelne Mitarbeiter erkrankt sind oder weil die Firmen einfach weniger verkaufen.“ (Nienhaus, Schieritz 2020, S. 21). Die Lieferketten nach Deutschland – aber auch von Deutschland zu den ausländischen Partnern – sind also gestört, wenn nicht zerbrochen. In China hat die Epidemie wohl ihren Ursprung, sie hat sich zu einer Pandemie entwickelt, die auch Europa hart getroffen hat, vom Rest der Welt erst gar nicht zu sprechen. Diese Katastrophe, in der es um Leben und Tod geht, hat die politischen Akteure alarmiert und veranlasst, das wirtschaftliche und gesellschaftliche Leben durch einen Shutdown still zu legen. Die Maßnahmen unterscheiden sich von Land zu Land, mal sind sie allumfassend und streng, mal weniger total und lockerer. Mit dieser Anordnung verbindet sich die Hoffnung, die Pandemie zu verlangsamen und so das Gesundheitssystem funktionstüchtig zu halten. Nur wenige lebensnotwendige Bereiche wie Supermärkte und Bäckereien, Apotheken und Drogerien blieben offen. Die Menschen sind gehalten, ihre Wohnungen nicht zu verlassen und den Kontakt zu anderen zu meiden. Regelverstöße werden staatlicherseits geahndet. Durch diese Maßnahmen gingen die Ansteckungs- und Todeszahlen nach unten, sodass schrittweise das Erwerbs- und Alltagsleben wieder in Gang kommen kann. Darüber hinaus hat der Deutsche Bundestag in einer noch nie dagewesenen Höhe Hilfsprogramme für Solo-Selbstständige, Unternehmen und ArbeitnehmerInnen verabschiedet, um den wirtschaftlichen Niedergang zu bremsen. Weitere Stützungsmaßnahmen sind nicht ausgeschlossen. Der Kampf gegen das Virus, der das Wirtschaftswachstum blockiert, wird, wenn er in absehbarer Zeit durch die Entwicklung eines Impfstoffs hoffentlich gewonnen ist, durch ein Engagement für den Wiederaufbau der Volkswirtschaft ersetzt werden müssen. Möglicherweise muss der Kampf aber auch an zwei Fronten gleichzeitig geführt werden. Anzumerken ist, dass – wie bereits oben erwähnt – der Umgang der einzelnen Länder mit der Pandemie höchst unterschiedlich ist, zumal die Wirtschaftskraft divergiert, generell das Erfahrungswissen – abgesehen von medizinischen Experten – äußerst gering ist und das Handeln der Akteure durch spezifische politische Konstellationen und sozio-kulturelle Besonderheiten mitbestimmt wird.

Die dysfunktionalen Folgen der Verschlankung zeigen sich auf vielfältige Weise: Als Folge des *Personalabbaus,* durchaus auf unterschiedliche Ursachen zurück zu führen, wurden die Unternehmen bzw. Organisationen schlank bis zur „Magersucht“, sodass Beschäftigung wieder aufgebaut werden musste. Im Gesundheitssystem, auf das noch genauer einzugehen ist, ist der Personalmangel, vor allem bei den Pflegekräften, ein Gutteil auf die prekären Arbeitsbedingungen zurückzuführen: Das Defizit bedeutet für die verbliebenen Beschäftigten eine Arbeitsintensivierung verbunden mit erheblichen Überstunden bei einem gleichzeitig geringen Entgelt, was weitere Kündigungen und sogar frühzeitigen Verschleiß mit Ausscheiden aus dem Beruf begünstigt sowie die Gewinnung von Nachwuchskräften erschwert.

Auch die anderen, bereits vorgestellten Methoden erweisen sich als dysfunktional: *Outsourcing*, das national wie international aus Kostengründen erfolgt(e), vernachlässigte anfangs die Produktqualität. Sie erreicht(e) vielfach nicht das erforderliche Niveau, sodass Schulungen notwendig sind, um einen Null-Fehler-Standard zu erreichen. Ferner wird mit insourcing, also einer Rückführung reagiert. Aber eine Umkehr des Trends auf breiter Front wird es kaum geben, denn die internationale Arbeitsteilung hat sich ständig ausgeweitet und vertieft, zumal die äußerst geringen Transportkosten, etwa mit Containerschiffen, ein zu vernachlässigender Faktor sind. Es werden also transnationale Wertschöpfungsketten weiterhin existieren und neu aufgebaut werden. Allerdings findet parallel eine Renationalisierung statt, sie begann bereits als Reaktion auf die Finanzkrise, auf den Brexit und auf die Politik „America first!“. Überdies müssen nationale Unternehmen auch international präsent sein, um ihre Marktchancen global besser nutzen zu können. Ferner kaufen sich ausländische Unternehmen in nationale ein, sie werden Teilhaber oder erwerben die Firma gleich ganz. Mit Blick auf China zeigt sich mittlerweile eine deutliche Zurückhaltung von Seiten der deutschen Industrie und Politik. Vermutlich gilt auch für die Zukunft: Outsourcing erfolgt weiterhin aus Kostengründen, sodass die *kostengünstigste Beschaffung von (Teil)Produkten* avisiert wird. Die Unternehmen, die möglichst nur* mit einem Lieferanten *zu tun haben wollen, versprechen sich davon eine weitere Optimierung ihrer Wirtschaftsbeziehungen. Jedoch nehmen sie dabei eine fragile Lieferkette in Kauf. Eine nach dem* Prinzip just-in-time* organisierte Fertigung erweist sich ebenfalls als störanfällig, etwa durch schlechtes Wetter, Staus auf den Straßen, Streiks in den ausländischen Zulieferbetrieben, so dass in gewissem Umfang wieder Lager aufgebaut werden mussten und müssen.

Blicken wir nun auf das Gesundheitssystem in der Bundesrepublik, wobei sich vergleichbare Tendenzen auch in anderen Ländern zeigen: Zwar haben WissenschaftlerInnen schon vor Jahren vor einer Pandemie gewarnt und Vorräte an Betten sowie zusätzliche Ausrüstung in Krankenhäusern, Pflegeheimen, ambulanten Pflegediensten, Arztpraxen angemahnt, aber dazu ist es zumindest nicht in ausreichendem Maße gekommen (vgl. Mascolo und Richter 2020, S. 2; Mayr 2020, S. 3). Durch* outsourcing* wurde die Bundesrepublik – einst „die Apotheke der Welt“ – im Gesundheitssystem abhängig von Indien und China, und zwar vor allem bei Arzneimitteln und Wirkstoffen, aber auch bei Schutzmaterial für die Beschäftigten (vgl. Malcher 2020, S. 21). Die bis vor kurzem auffällige Ignoranz rächt sich nun. Die defizitäre Entwicklung hat zwar mit dem Versagen von Schlüsselpersonen zu tun, aber es gibt dafür tiefere, nämlich die skizzierten systemischen Ursachen, die das Handeln der Verantwortlichen leiten: Mit der neoklassischen bzw. neoliberalen Wirtschaftspolitik rückt der Markt als Problemlöser zu Lasten staatlichen Handelns in den Fokus, und infolge dessen orientieren sich die Akteure im Bereich öffentlicher Dienstleistungen am Lean-Leitbild. Diese Strategie, unter dem Label New Public Management bekannt geworden, hat sich zunehmend durchgesetzt (vgl. Naschold 2000). Krankenhäuser und Pflegeheime sind ein offenkundiges Beispiel dafür, wie öffentliche Dienstleistungen nach betriebswirtschaftlichen Kennziffern umorganisiert wurden und werden, sodass den geforderten – früher unbekannten – Gewinnerwartungen entsprochen werden kann. Gemäß dieser Managementlehre wurden und werden kostenintensive Bereiche wie das Personal und die medizinischen Ressourcen abgebaut, überdies erfolgt(e) – zumindest teilweise – eine Privatisierung. Der in der Corona-Katastrophe gravierende Mangel, beispielsweise an Schutzkleidung, Gesichtsmasken, Beatmungsgeräten, Medikamenten und Rohstoffen für Pharmazeutika, ist – wie bereits vermerkt – ein Resultat der zunehmenden Internationalisierung der Produktion seit den neunziger Jahren.* Die Lieferung just-in-time, häufig nur von einem Produzenten*, funktioniert nur solange wie die Lieferketten stabil sind, was im Moment nicht mehr der Fall ist. Zusammen mit der mangelnden Vorratshaltung erreicht das Defizit an medizinischem Material ein gigantisches Ausmaß. In der Krise kommt es daher zu einer, vermutlich temporären Konversion in der nationalen Produktion: Beispielsweise haben Unternehmen der Textilbranche, die seit den siebziger Jahren des vergangenen Jahrhunderts zunächst nach Südeuropa und anschließend nach Asien* outgesourct* wurden und die folglich kaum noch in Deutschland präsent sind, ihre Fertigung auf medizinisch notwendigen Produkte, etwa Gesichtsmasken, umgestellt. Weitere Unternehmen der Schlüsselindustrien wie Maschinenbau, Automobil und Chemie zeigen sich für eine Konversion ebenfalls offen. Damit versuchen sie, ihre aktuellen Umsatzeinbußen auszugleichen und signalisieren zugleich ihre patriotische Verbundenheit. Andere Unternehmen nutzen ihre weltweiten Beziehungen, um Schutzmaterial im Auftrag der Regierung zu kaufen. Sie alle tragen zur Behebung der Mangelwirtschaft bei, was allemal mehr ist als nur ein symbolischer Beitrag.

Die Pandemie, die die desaströsen Mängel im Gesundheitssystem und in der Versorgung offen legt, erzwingt eine Revision bisheriger Praxis, denn diese gefährdet die Sicherheit der Menschen fundamental. Mehr noch – das zerstörerische Potential dieser Krise beschädigt letztlich alle gesellschaftlichen Bereiche. Es ist keineswegs auszuschließen, dass sich eine solche verheerende Naturkatastrophe zukünftig wiederholt.

Mit der Ökonomisierung des Gesundheitssystems einschließlich der skizzierten Folgen wird eine verlässliche Daseinsvorsorge und Fürsorge gefährdet, die öffentliche Dienstleistungen garantieren sollen. In der gesellschaftlichen Debatte wird bereits jetzt, mitten in der Krise eine Rückführung, ein insourcing von „systemrelevanten“ Produkten einschließlich der Fertigung in die nationale bzw. europäische Wirtschaft gefordert, um die fragile globale Abhängigkeit zu mildern und lebensnotwendige Ressourcen zu sichern. Die Akteure in der Politik diskutieren zudem wirtschaftspolitische Instrumente, die eine solche Umkehr staatlicherseits unterstützen könnten. Wie weit der Prozess der Deglobalisierung gehen wird, der längst vor der Pandemie begonnen hat, lässt sich nicht voraussagen. Die Digitalisierung, die sich nach Meinung von Fachleuten in und nach der Krise beschleunigen wird, könnte die Renationalisierung antreiben, denn bei der damit einhergehenden Automatisierung spielen Lohnkosten so gut wie keine Rolle mehr. Unter dem Deckmantel der Rezession wird also vermutlich die Rationalisierung als Automatisierung forciert. Zu befürchten sind dabei negative Effekte für die Beschäftigung. Diese dürften bei einem technikzentrierten Ansatz, der als Mainstream gelten kann, größer sein als bei einem arbeitsorientierten, der die Arbeit durch Digitalisierung unterstützen und nicht eliminieren, also automatisieren will (vgl. Raehlmann 2019). Eine Abkehr von der Globalisierung wird allerdings, wie bereits angemerkt, nicht stattfinden, jedoch werden bestimmte Maßnahmen weiter korrigiert werden müssen, etwa das Ausmaß von outsourcing und dabei die Abhängigkeit von nur einem Produzenten sowie schließlich der Umfang der just-in-time Produktion.

Mit den organisatorischen Korrekturen sind auch Verbesserungen der Arbeitsbedingungen erforderlich, was für das medizinische Personal, aber besonders für die pflegenden Arbeitskräfte gilt. Solche Anstrengungen sind zudem geboten, um den Fachkräftemangel dauerhaft zu beheben. Die Wertschätzung in der Pandemie sollte sich auch beim Entgelt zeigen. Eine komplette Automatisierung der personenbezogenen Dienstleistung ist nicht möglich, sie verbietet sich schon aus ethischen Gründen. Gleichwohl ist eine Unterstützung dieser Arbeit durch Digitalisierung möglich und sinnvoll. Daher bleibt das Thema Entgelt aktuell. Das Entgelt ist aus historischen wie systematischen und nicht zuletzt aus geschlechtsspezifischen Gründen bis heute niedrig (vgl. Raehlmann 2013). Die Forderung nach einer Erhöhung lässt sich nicht so leicht umsetzen, wie vielfach suggeriert, denn Entgelte – wie die Gesamtheit der Arbeitsbedingungen – sind „Kampf- und Kompromissprodukte, also Erzeugnisse von Machtkonstellationen“ (Weber 1921/1964, S. 77). Tarifverträge sind dafür eine unverzichtbare Voraussetzung. Die Mitgliedschaft des Pflegepersonals in den Gewerkschaften ist gering und folglich auch ihre Konfliktfähigkeit mit den Arbeitgebern. Es kommt noch ein weiterer Sachverhalt hinzu: Die beiden Kirchen sind mit über einer Million Beschäftigten in ihren Einrichtungen der größte Arbeitgeber. Das kirchliche Arbeitsrecht, eine deutsche Besonderheit, kennt keinen Betriebsrat, kein Streikrecht und keine Teilnahme an Tarifverhandlungen, sodass die Arbeitskräfte wohl kaum motiviert sind, einer Gewerkschaft beizutreten. Mit anderen Worten: Die Verhandlungsmacht der Beschäftigten und ihrer Organisationen ist schwach. Das sind in der Summe wichtige Faktoren, die das aktuell niedrige Entgelt zu erklären vermögen.

## Plädoyer für ein erweitertes Verständnis von Rationalisierung

Die praktischen, weltweiten Erfahrungen mit lean production/lean administration, die auf Grund ihrer ambivalenten Wirkungen Korrekturen erfordern, halten noch eine Botschaft für die ArbeitswissenschaftlerInnen bereit. Sie ist zwar nicht gänzlich neu, aber in der Pandemie zeigt sich ihre Aktualität. Seit ihren Anfängen thematisiert die Arbeitswissenschaft den Prozess der Rationalisierung, allerdings vornehmlich als betriebliches Geschehen, als Kooperation zwischen Arbeitgeber bzw. Management und Arbeitskraft, wobei volkswirtschaftliche Bezüge eher unterbelichtet bleiben. Diese einzelwirtschaftliche Sicht war und ist unverzichtbar, aber zu eng:

Manche der früheren Defizite der Arbeitsbedingungen sind durch die Entwicklung des Sozialstaats, so durch gesetzliche Bestimmungen und, soweit existent, durch Tarifverträge gemildert, wenn nicht gar beseitigt worden. Jedoch müssen die sozialen Standards immer wieder verteidigt werden. Die Verbesserungen gelten aber kaum für Unternehmen der* Entwicklungs- und Schwellenländer, den sogenannten Billig-Lohn-Ländern*, wo der Arbeits- und Gesundheitsschutz – häufig erst nach betrieblichen Unfällen bis hin zu Katastrophen – eingefordert wird. Die diesbezüglichen Unternehmen sind die bevorzugten Adressaten der Politik des Outsourcing. Über die klassische Interessenorientierung hinaus sind die Interessen dieser Länder und nicht zuletzt diejenigen der *Länder der EU* ebenfalls in den Blick zu nehmen. In der Pandemie zeigt sich, wie sehr* die nationale bzw. europäische Sicherheit* durch Outsourcing im Gesundheitssektor bedroht werden kann, sodass potentielle Auslagerungen prinzipiell der Überprüfung bedürfen. In den Debatten spielen überdies ö*kologische Fragen* eine immer stärkere Rolle. Es geht um umweltverträgliche Produktionsverfahren und Produkte, um den Klimawandel in Schach zu halten und auf dem Globus akzeptable Lebensbedingungen für Mensch, Tier und Natur erhalten zu können bzw. wieder herzustellen. Ein Prinzip, das schließlich diese hoch dynamischen und divergenten Interessenlagen sowie -konflikte überwölben sollte, stellt die *Orientierung an Nachhaltigkeit* dar. Die Ergänzungen bedeuten eine deutliche Komplexitätssteigerung. Optimismus hinsichtlich der praktischen Umsetzung dieser Leitlinien durch diverse (politische) Akteure ist unangebracht, ganz im Gegenteil: Es handelt sich um ein äußerst schwieriges, gleichwohl notwendiges Unterfangen. Dabei kann der Beitrag der Arbeitswissenschaft darin bestehen, die Perspektive von Rationalisierung im Sinne genannter Prinzipien zu erweitern, d. h. die damit verbundene Vielfalt von Interessen, Konflikten und Spannungsfeldern klar zu benennen und zu thematisieren.
